# Benign hibernoma mimicking a cardiac liposarcoma

**DOI:** 10.1093/jscr/rjae510

**Published:** 2024-09-05

**Authors:** Kody Wyant, Titilayo Oden Shobayo, Manuel R Rojo, Zaid M Abdelsattar, Menhel Kinno, Jeffrey Schwartz

**Affiliations:** Stritch School of Medicine, Loyola University Chicago, Maywood, IL 60153, United States; Department of Thoracic & Cardiovascular Surgery, Loyola University Medical Center, Maywood, IL 60153, United States; Stritch School of Medicine, Loyola University Chicago, Maywood, IL 60153, United States; Stritch School of Medicine, Loyola University Chicago, Maywood, IL 60153, United States; Department of Thoracic & Cardiovascular Surgery, Loyola University Medical Center, Maywood, IL 60153, United States; US Department of Veterans Affairs, Edward Hines Jr. VA Hospital, Hines, IL 60141, United States; Stritch School of Medicine, Loyola University Chicago, Maywood, IL 60153, United States; Department of Thoracic & Cardiovascular Surgery, Loyola University Medical Center, Maywood, IL 60153, United States; US Department of Veterans Affairs, Edward Hines Jr. VA Hospital, Hines, IL 60141, United States; Stritch School of Medicine, Loyola University Chicago, Maywood, IL 60153, United States; Department of Thoracic & Cardiovascular Surgery, Loyola University Medical Center, Maywood, IL 60153, United States; Stritch School of Medicine, Loyola University Chicago, Maywood, IL 60153, United States; Department of Thoracic & Cardiovascular Surgery, Loyola University Medical Center, Maywood, IL 60153, United States

**Keywords:** hibernoma, cardiac tumor, rare, benign

## Abstract

Despite the low incidence of primary cardiac tumors, recently at our institution, we have experienced two very rare tumors in the span of just a few months. Hibernomas are rare tumors of brown adipose tissue origin that share the benign clinical features of a lipoma, but on imaging mimic the more aggressive sarcoma. Here we present two separate cases of otherwise healthy patients who were found incidentally to have these asymptomatic tumors.

## Introduction

The incidence of primary cardiac tumors is extremely low at <1%. Of those, the majority are benign, with myxoma being the most common. Only one quarter of primary cardiac tumors are malignant, of which cardiac sarcomas are the most common [1]. Preoperative imaging with echocardiography and magnetic resonance imaging usually provides sufficient information on the location, functional features, and soft-tissue characterization to distinguish benign from malignant pathology. In the absence of metastatic disease, complete surgical resection is the goal.

In this case series, we present two cases where a rare benign tumor mimicked the preoperative features of cardiac sarcoma.

## Case report A

An otherwise healthy 69-year-old female was undergoing workup for total knee arthroplasty when she was referred to a cardiologist for symptoms of dizziness. Her symptoms were infrequent and non-reproducible. She denied any chest discomfort, shortness of breath, orthopnea, facial swelling, or peripheral edema. Her physical exam and laboratory workup were unremarkable.

**Figure 1:**
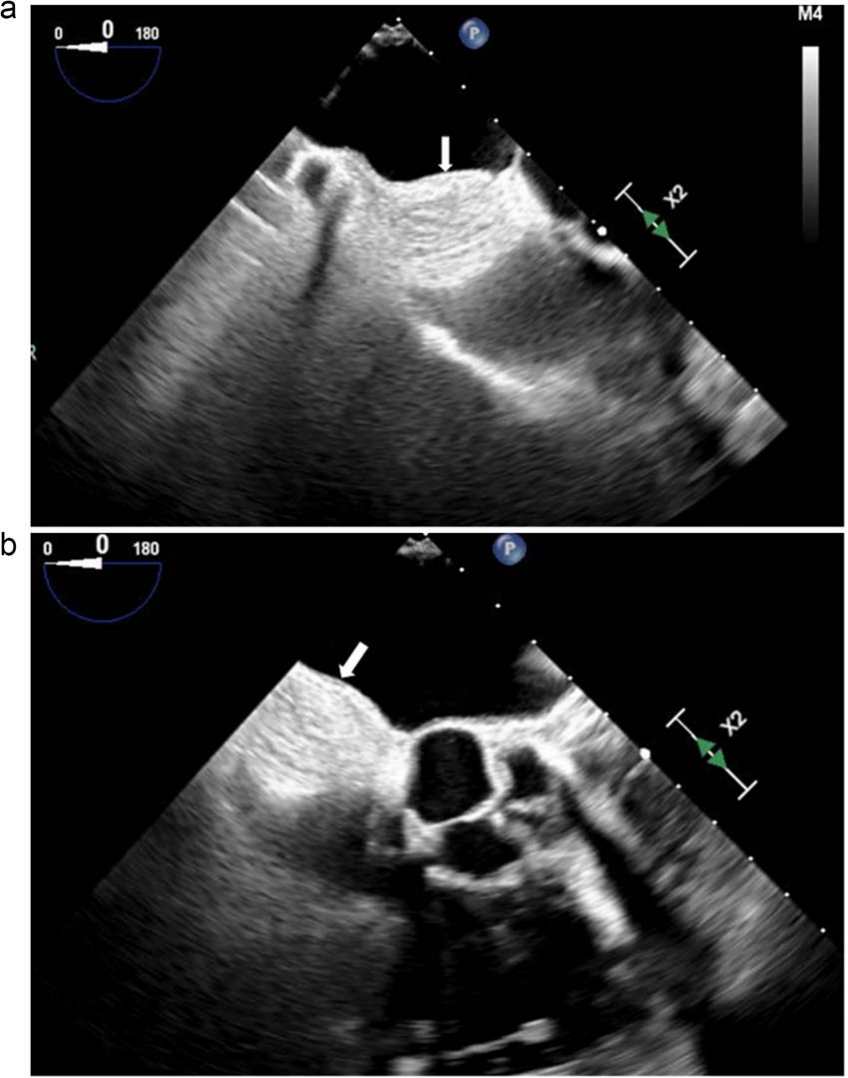
(a and b) Transesophageal echocardiogram. Arrow denotes mass involving the interatrial septum.

**Figure 2:**
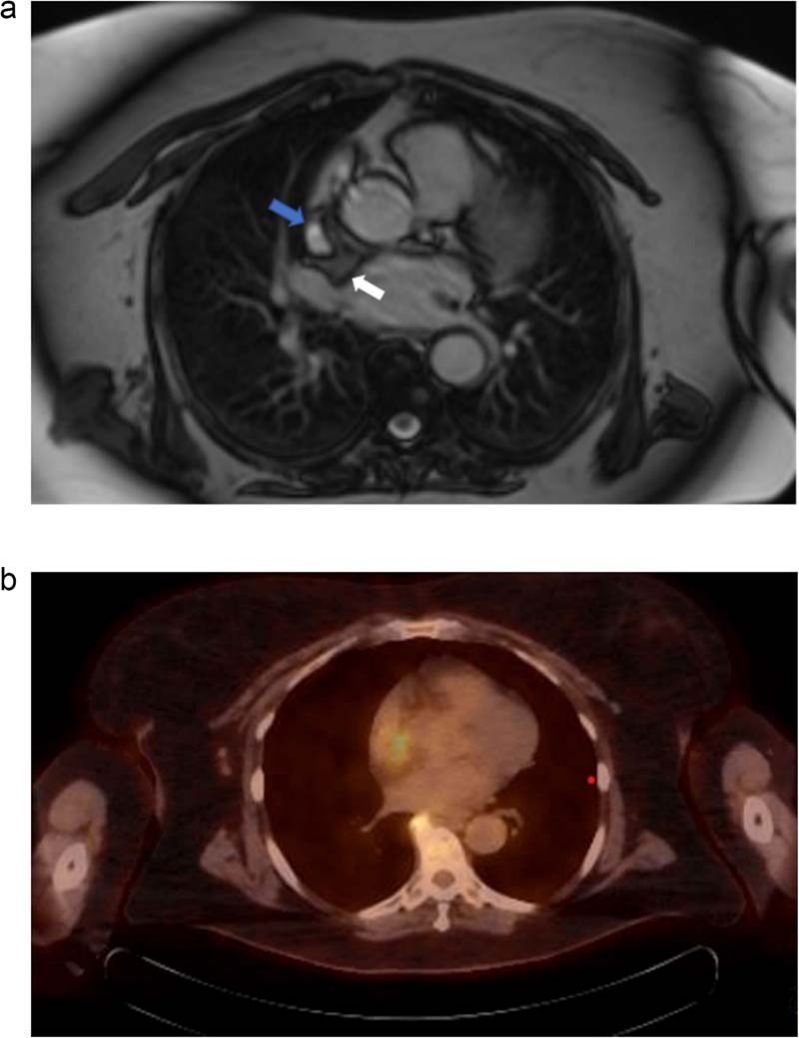
(a) Cardiac MRI. Mass is represented by the arrow on the right. The left arrow demonstrates the SVC. (b) PET/CT. Uptake is demonstrated in the right atrium. Uptake near the spine was determined to be artifact and not representative of metastatic disease.

Electrocardiography revealed normal sinus rhythm. An echocardiogram was performed, which revealed a mass involving the interatrial septum extending superiorly toward the superior vena cava (Fig. 1). She had normal biventricular function with a left ventricular ejection fraction of 55%, no valvular abnormalities, and no evidence of obstruction. Subsequently, cardiac magnetic resonance imaging (MRI) was performed. This showed a 5.5 cm x 2.5 cm x 2.5 cm mass involving the interatrial septum with near encasement of the superior vena cava (Fig. 1). The mass extended inferiorly toward the inferior vena cava (IVC) and coronary sinus, but without obvious involvement. The mass was hyperintense on T1 and T2-weighted sequences. On T1 fat saturating images, it was hypointense. It showed contrast enhancement with gadolinium suggesting vascularity. The staging workup further included positron emission tomography (PET), which showed increased fluorodeoxyglucose-18 (FDG) uptake in the atrial tumor without evidence of extracardiac disease or metastases (Fig. 2b). These findings were suggestive of a primary cardiac liposarcoma. The patient was then referred to our clinic for consideration of resection. She was deemed an operable candidate and the tumor resectable.

The operation was performed via a standard median sternotomy with aorto-bicaval cannulation. The superior vena cava (SVC) was cannulated high, and the right femoral vein was cannulated to allow for possible resection of the cavae ensuring resection of all positive margins and a tumor-free reconstruction. The right atrium was opened, and the mass was readily palpable; although visually, it was indistinguishable from the normal endocardium. The palpable firmness helped guide the resection. The entire interatrial septum was resected; anteriorly within 1 cm of the AV groove, superiorly past the level of the atriocaval junction, and inferiorly, directly adjacent to the coronary sinus and IVC. The mass was oriented and sent for frozen section. Frozen section was suspicious for liposarcoma but not conclusive, with a positive margin inferiorly, near the coronary sinus. Further resection at this margin could not have been possible without compromise of the coronary sinus orifice, thus we accepted the possible positive margin and proceeded with reconstruction. Reconstruction of the interatrial septum was completed with a standard single patch technique using bovine pericardium. An additional bovine pericardial patch was used to reconstruct the anterior wall of the right atrium as well as the SVC and IVC. The patient was weaned from cardiopulmonary bypass and decannulated without complication. The patient was ultimately discharged to acute inpatient rehabilitation on postoperative Day 17 with no residual symptoms. She has since made a complete recovery and has been able to undergo total knee arthroplasty.

Final pathologic review showed eosinophilic, polygonal, and multivacuolated granular brown fat cells with variable components of univacuolated white fat. There were no prominent atypia or atypical mitotic figures identified. These histopathologic findings are consistent with a hibernoma rather than a liposarcoma. Additional molecular studies for murine double minute 2 gene, a known E3 ubiquitin ligase and negative regulator of the tumor suppressor gene p53, which is usually present in liposarcomas were negative, further supporting the diagnosis of hibernoma.

## Case report B

**Figure 3:**
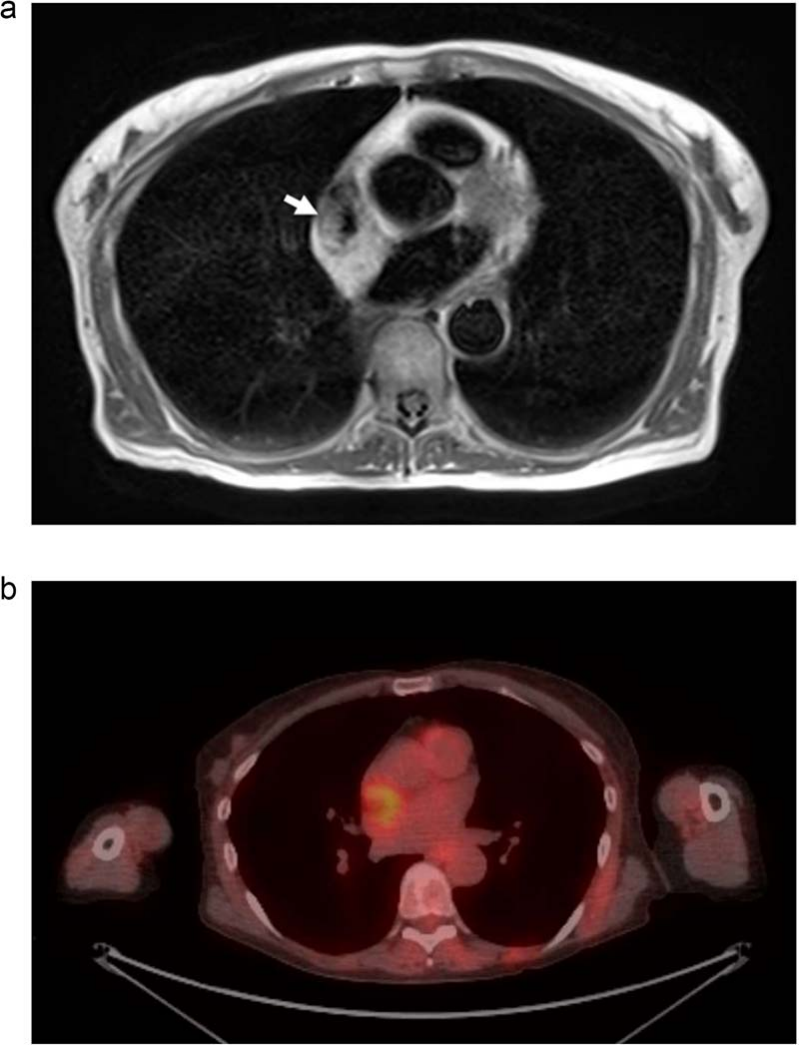
(a) MRI chest. Mass represented by white arrow. (b) PET/CT. Uptake is demonstrated in the mass associated with the SVC and right atrium.

We present another rare case of a hibernoma occurring just a few months later. A 63-year-old woman with history significant for HTN, COPD, GERD, ETOH abuse, and a 54-pack year tobacco use experienced continued weight loss of 70 lbs in the last 5 years. She underwent extensive workup for malignancy, which included MRI of the chest. MRI showed a 3.0 x 3.1 cm tumor circumferentially involving the superior vena cava at the atriocaval junction. This mass was hyperintense on T1 and T2-weighted images (Fig. 3a). Transesophageal echocardiogram was performed, and the mass was not appreciated. PET showed increased FDG uptake in the mass area without evidence of extracardiac disease (Fig. 3b). The recommendation was made for surgical resection.

The operation was performed via standard median sternotomy with aorto-bicaval cannulation for cardiopulmonary bypass. Circumferential dissection and control of the SVC were obtained just below the junction of the azygous vein. There was a large pretracheal lymph node, which was excised and sent for frozen pathology; it was benign. A right atriotomy was made and the mass could be palpated within the proximal right atrium at the right atrial-SVC junction and extending into the interatrial septum just above the fossa ovalis. The incision extended medially across the right atrial appendage medially to the interatrial septum, which was then entered, leaving a rim of right atrium and interatrial septum for reconstruction. The interatrial septum was excised just down to the top of the fossa ovalis and then laterally to the origin of the right superior pulmonary vein. The SVC was transected ~2–3 cm above the right atrial SVC junction. A bovine pericardial patch was then used to repair the interatrial septum. The SVC was reconstructed with a bovine pericardium, which was tailored into a tube using a 36-French chest tube and then anastomosed to the SVC. This specimen was marked for orientation and sent for frozen section, which showed no evidence of malignancy. Final pathologic diagnosis confirmed a benign hibernoma.

The patent’s postoperative course was complicated by symptomatic junctional bradycardia. Dual chamber permanent pacemaker was implanted 8 days postoperatively. She was discharged home on postoperative Day 10.

## Discussion

Hibernomas are rare tumors of brown adipose tissue origin. They are benign lipomatous neoplasms with no malignant potential and often occur in the soft tissues of the thigh, trunk, or chest region. This is one of very few reported cases of cardiac hibernoma. First described in 1906, these tumors are similar to lipomas in clinical behavior but have unique imaging and histopathologic features [2]. Their histopathology is characterized by large multi-vacuolated cells with an abundance of adipose. This typical subtype accounts for 82% of cases; however, lipoma-like, myxoid, and spindle cell variants have been described [3].

Differentiating hibernomas from other fat-containing tumors is difficult, as imaging alone is not sufficient to support a definitive diagnosis. On echocardiography, hibernomas are encapsulated and well-circumscribed. They have variable prominent vessels or vascularity, which are features shared with typical and atypical lipomas. MRI shows heterogeneous signal enhancement in both T1 and T2-weighted sequences, as well as gadolinium contrast enhancement [4]. These features make distinguishing a hibernoma from a liposarcoma difficult. PET imaging will show FDG activity owing to the high metabolic activity of brown fat.

Surgical excision is the only strategy to definitively diagnose and treat these neoplasms. Given the benign nature of these neoplasms, prognosis is excellent, and recurrence should be rare [5]. Both patients will have follow-up with cardiac MRI at 6 months postoperatively to establish new baseline imaging. Disease recurrence or progression.
